# Ultrasound-Assisted Encapsulation of Anthraquinones Extracted from Aloe-Vera Plant into Casein Micelles

**DOI:** 10.3390/gels8090597

**Published:** 2022-09-17

**Authors:** Uzma Sadiq, Harsharn Gill, Jayani Chandrapala

**Affiliations:** School of Science, RMIT University, Bundoora, Melbourne, VIC 3083, Australia

**Keywords:** casein micelles, ultrasonication, nanoencapsulation, *Aloe vera* extracted anthraquinones, spray dried powder, freeze-dried powder, aloin, aloe-emodin, rhein

## Abstract

*Aloe-vera* extracted anthraquinones (aloin, aloe-emodin, rhein) possess a wide range of biological activities, have poor solubility and are sensitive to processing conditions. This work investigated the ultrasound-assisted encapsulation of these extracted anthraquinones (AQ) into casein micelles (CM). The particle size and zeta potential of casein micelles loaded with aloin (CMA), aloe-emodin (CMAE), rhein (CMR) and anthraquinone powder (CMAQ) ranged between 171–179 nm and −23 to −17 mV. The AQ powder had the maximum encapsulation efficiency (EE%) (aloin 99%, aloe-emodin 98% and rhein 100%) and encapsulation yield, while the whole leaf *Aloe vera* gel (WLAG) had the least encapsulation efficiency. Spray-dried powder (SDP) and freeze-dried powder (FDP) of *Aloe vera* showed a significant increase in size and zeta potential related to superficial coating instead of encapsulation. The significant variability in size, zeta potential and EE% were related to anthraquinone type, its binding affinity, and its ratio to CM. FTIR spectra confirmed that the structure of the casein micelle remained unchanged with the binding of anthraquinones except in casein micelles loaded with whole-leaf aloe vera gel (CMWLAG), where the structure was deformed. Based on our findings, *Aloe vera* extracted anthraquinones powder (AQ) possessed the best encapsulation efficiency within casein micelles without affecting its structure. Overall, this study provides new insights into developing new product formulations through better utilization of exceptional properties of casein micelles.

## 1. Introduction

Identification and extraction of biologically active components from plants have led to the discovery of a range of nutraceuticals, pharmaceuticals and functional foods for health improvement [1]. There are a total of 21,000 plants used for medicinal purposes throughout the world, out of which <200 have been used commercially by biopharmaceutical companies [2]. *Aloe vera* is a magical plant in Ayurveda that contains plenty of secondary metabolites [3]. Secondary metabolites of *Aloe vera* are the anthraquinones, including aloin, aloe-emodin and rhein, that are tricyclic aromatic quinones produced through a plant-specific type III polyketide biosynthesis pathway [4]. These anthraquinones possess a wide range of biological and pharmacological activities. These include laxatives [5], anticancer [6,7,8,9], antibacterial [10,11], anti-inflammation, homeostatic and antispasmodic [12]. Although *Aloe vera* has been used in many food systems such as ready-to-serve drinks, soft drinks, yoghurt, sports drinks, laxative drinks, whisky and white bread [13] for decades, the available information concerning the effect of food processing on the degradation of anthraquinones during preparation is limited.

In our previous study, it was reported that the degradation of these anthraquinones starts right after harvesting the *Aloe vera* leaves and continues to decompose during processing and storage [14]. So, encapsulating these anthraquinones seems to be an efficient method for food formulators while developing traditional medicines, functional foods, and nutraceuticals. In addition, the low bioavailability and instability of anthraquinones during digestion greatly limit their health benefits. So it is extremely important to have a protective mechanism capable of preserving these anthraquinones’ chemical integrity while delivering their physiological benefits [15].

Encapsulation increases the stability of bioactive components while preserving their functional potential. The encapsulation procedure traps an active substance (solid, liquid or gas) in a polymeric matrix or wall to protect the compound from environmental conditions and interactions with other components or control its release in foods or consumers [16]. So, the selection of wall material is of great importance for encapsulation. Casein micelles (CM) possess unique structural and physicochemical properties for the protection and transport of bioactive components [17,18]. Moreover, casein micelle’s hydrophilic and hydrophobic domains possess the capability to interact with various bioactives. The exceptional stability of casein micelles during heating, freezing and drying processing make them ideal for encapsulating bioactive components. CM’s excellent self-assembling, emulsifying, gelation and higher water-binding abilities make them an appropriate wall material for encapsulation [19]. Additionally, casein micelles are recognized as GRAS (Generally Recognized as Safe) [20], readily available and less expensive than milk protein monomers, such as β casein, β lactoglobulin and other proteins.

Previous studies showed the use of casein micelles (CM) to encapsulate β carotene [21], curcumin [22,23] and vitamin A, D_2_ and D_3_ [24,25]. Most recently, Nascimento and his colleagues used CM to encapsulate phenolics from Jaboticaba fruit [26] at various pH conditions (2.0, 7.0) by using transglutaminase (TG). They reported 70% degradation of phenolic compounds during drying irrespective of pH. Curcumin, a polyphenol extracted from turmeric, has been encapsulated successfully (97%) into casein micelles without affecting the techno-functional properties of CM [27]. Researchers [28] used re-assembled CM to encapsulate quercetin (lipophilic flavonoid), a therapeutic agent extracted from dry onion peel, to enhance its water solubility and bioavailability. Yan and colleagues [29] microencapsulated vitamin C using renneted casein micelles followed by spray drying. During storage over 10 weeks, the micro capsulated gummies retained more vitamin C (92%) than encapsulated gummies (79%). Moreover, encapsulated gummies showed better protection and a slower release rate in gastric and intestinal fluids.

Various encapsulation technologies, including spray drying, freeze drying, and coacervation, have previously been used to protect *Aloe vera* components from degradation using different wall materials. However, ultrasonic encapsulation offers improved energy efficiency, emulsion stability, reduced surfactant usage, and controlled size distributions [30]. Most recently, ultrasonic encapsulation of α-Tocopherol (lipophilic compound) into casein nanoparticles [31] reported a change in the secondary structure of casein micelles by the binding of α-tocopherol as well as a decrease in the ratio of α-helix to the β-sheet structure of CM. Encapsulation of emodin (a natural polyphenol) into micellar casein under heated conditions resulted in three times higher binding constants with ultrasound [32] compared to un sonicated samples. In addition, ultrasonication slowed the release of emodin in gastric and intestinal fluids during digestion.

Although much work has been carried out regarding casein micelles as a delivery system for pharmaceuticals and nutraceuticals [26,32,33,34], there is hardly any work on the ultrasound-assisted encapsulation of anthraquinones extracted from *Aloe vera* into casein micelles. Therefore, the present study evaluated the use of ultrasound to encapsulate anthraquinones (aloin, aloe-emodin, rhein) extracted from the *Aloe vera* plant within casein micelles. The anthraquinone encapsulated casein micelles were characterized for their particle size, zeta potential, encapsulation efficiency and encapsulation yield. Furthermore, FTIR was performed to assess the interaction among anthraquinones and casein micelles. The successful encapsulation of anthraquinones into casein micelles would increase the potential of using anthraquinones in many thermally treated food products.

## 2. Results and Discussion

### 2.1. Quantification of Anthraquinones

**Figure 1:**
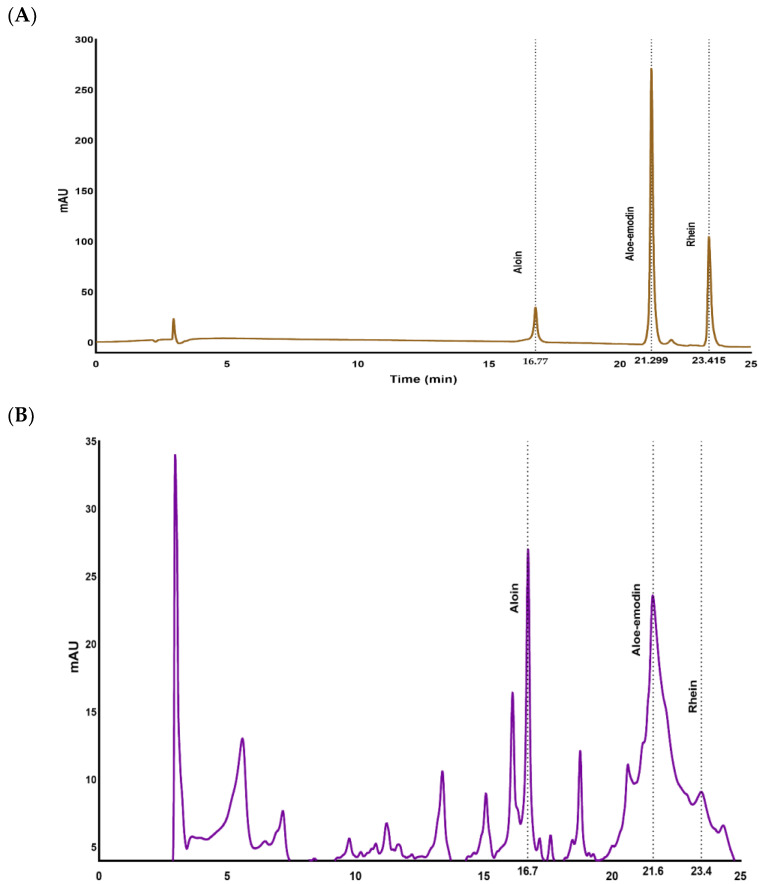
HPLC chromatographs of (**A**) reference standards of aloin, aloe-emodin and rhein and (**B**) HPLC chromatograms of extracted anthraquinone powder (AQ) from Aloe vera containing aloin, aloe-emodin and rhein.

Figure 1A,B show the baseline separation of a mixture of the three anthraquinone standards and anthraquinones present in the AQ powdered sample respectively. Robust baseline separation was achieved in 25 min with retention times of 16.74, 21.21 and 23.40 for aloin, aloe-emodin and rhein, respectively.

Table 1 shows the concentrations of anthraquinones (aloin, aloe-emodin, rhein) initially present in whole leaf Aloe vera gel (WLAG), extracted anthraquinone powder (AQ), spray dried powder (SDP) and freeze-dried powder (FDP) of Aloe vera determined through HPLC. Aloin concentration was the highest in all samples followed by aloe-emodin and rhein. The lower amounts of anthraquinones in FDP and SDP were due to the failure to preserve phenolic glycosides, inefficient solvent extraction, and exposure of polyphenols to high temperatures during spray drying as reported by Uzma et al. (2022) (14). WLAG, FDP and SDP showed no rhein present which most likely is due to the low content of aloin to start with which was not enough to be oxidized to rhein (14).

### 2.2. Encapsulation Efficiency and Yield of CM-Loaded with Anthraquinones

**Figure 2:**
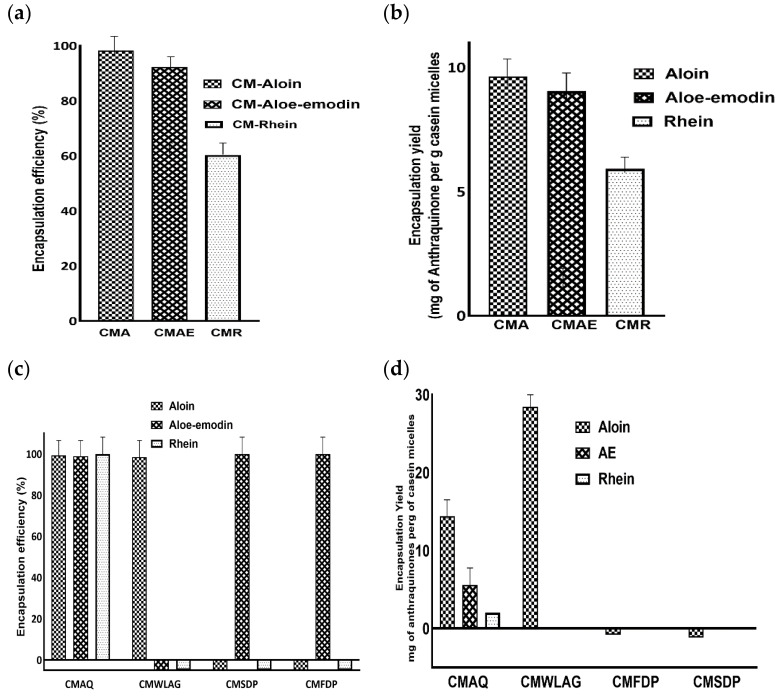
Plot of encapsulation efficiency (EE%) and encapsulation yield (EY) of casein micelles loaded with pure aloin, aloe-emodin, rhein (**a**,**b**) and encapsulation efficiency (EE%) and encapsulation yield (EY) of casein micelles after loading of Anthraquinones (aloin, aloe-emodin, rhein) from various samples of Aloe vera (AQ, FDP, SDP and WLAG) (**c**,**d**).

Encapsulation efficiency describes the ability of wall material to enclose the core compounds. The Encapsulation Efficiency (EE) of pure aloin, aloe-emodin and rhein within casein micelles ranged between 98% to 60% (Figure 2a). The higher encapsulation efficiencies of aloin (98%) and aloe-emodin (92%) were mainly due to the hydrophobic interactions between aromatic rings of aloin, aloe-emodin and hydrophobic amino acids (tyrosine and tryptophan) of casein micelles. Our results aligned well with the previous literature [35]. However, hydrogen bonding between the hydroxyl groups of aloin and aloe-emodin and the carboxyl groups of casein micelles [36] prevails. This was further highlighted by rhein’s encapsulation efficiency, which was 1.5 times lower than aloin and aloe-emodin.

Furthermore, aloin and aloe-emodin had higher encapsulation yields (9.6 and 9 mg per gram of casein micelles, respectively) compared to rhein (5.9 mg per gram of casein micelles), as shown in Figure 2b. This difference was primarily due to the capability of forming two hydrogen bonds by aloin and aloe-emodin while only one hydrogen bond can be formed by rhein with casein micelles [37]. Aloin and aloe-emodin contain two hydroxyl groups while only one hydroxyl group is present on ring C of rhein. Similarly, it was reported that increased hydroxylation on the C-ring of flavonoids resulted in the formation of more hydrogen bonds between flavonoids and polar groups of bovine serum albumin [38].

Encapsulation efficiency and encapsulation yield of anthraquinones from various *Aloe vera* samples (AQ, SDP, FDP, WLAG) are shown in Figure 2c,d, respectively. The highest encapsulation efficiency of anthraquinones (aloin 99%, aloe-emodin 98% and rhein 100%) was achieved by *Aloe vera* extracted anthraquinone powder (CMAQ). Similarly, AQ had the highest encapsulation yield for aloin (14.3 mg), aloe-emodin (5.5 mg) and rhein (1.1 mg) per gram of protein (Figure 2d). This high encapsulation efficiency corresponds to the easier access of aromatic rings and hydroxyl groups of anthraquinones present in AQ powders by aromatic amino acids and -NH or -OH groups of casein micelles in forming hydrophobic and hydrogen bonds respectively. In addition, a decrease in pH (7.4 to 5.5; Table 2) led to an increase in EE% by structural reorganization of casein micelles after binding anthraquinones during nanoencapsulation (CMAQ). The decrease in the negative charge of casein micelles at this pH value caused the CM to become more compact and retain more anthraquinones. These results are consistent with the previous literature that reported the highest encapsulation efficiency of β carotene at pH 5.5 [39,40].

*Aloe vera* spray-dried powder (SDP) and *Aloe vera* freeze-dried (FDP) powders showed zero encapsulation efficiency for aloin and 100% for aloe-emodin depending upon glycosylation. Aloin, as a glycoside, has a less binding affinity to casein micelles due to its hydrophilic nature and steric hindrance [41,42] while in powdered form. It has been reported previously [43] that increased glycosylation of polyphenols causes a decrease in the binding of polyphenols to milk proteins. A different trend was observed regarding the encapsulation of anthraquinones from WLAG (Figure 2c). The EE% was 98% for aloin due to its higher molecular weight (418.39) as compared to aloe-emodin (270.24) and rhein (284.22). Bulky compounds have more affinity to bind with proteins [44] due to more bond formations.

### 2.3. Particle Size and Zeta Potential for Colloidal Stability Evaluation

The average particle size of casein micelle samples before and after encapsulation of various anthraquinone sources extracted from *Aloe vera* is shown in Table 2. The size of casein micelles (CM) showed an insignificant reduction from ~178 nm to ~172–175 nm with the encapsulation of pure aloin (CMA), aloe-emodin (CMAE) and rhein (CMR) due to two reasons. Firstly, CM is a dense protein structure [45]. Ultrasonication facilitates the incorporation of anthraquinones (aloin, aloe-emodin, rhein) into the inner domain of casein micelles. It pulls casein chains closer [35], enhancing the interactions among casein micelles, anthraquinones and water (3.3 g/g of protein), as reported previously [46]. Secondly, the high proline content of casein micelles increased the binding propensity with polyphenols through hydrophobic and hydrogen bonding. However, in this study, the hydrophobic interaction might be generated among anthraquinones (aloin, aloe-emodin, rhein) and tyrosine, tryptophan or other amino acid residues close to tyrosine, tryptophan in casein micelles as reported previously [47]. The primary interaction between aromatic amino acids of the proteins and nonpolar aromatic rings of the polyphenols is hydrophobic [48]. Similarly to the current study, it has been reported that casein micelles interact with curcumin and cocoa polyphenols via hydrophobic and non-covalent interactions, respectively [49,50]. The decrease in CM size was aligned with previous work on CM encapsulation with DHA and jaboticaba extract [26,51].

The encapsulation of AQ powder insignificantly increased the casein micelle size by ~1 nm. The pH of the control casein micelle solution was 7.4, where increased intermolecular repulsions of individual caseins govern an enlarged and loosely packed micellar structure due to ionization of carboxylic groups [52,53]. This, in turn, causes exposure to hydrophobic domains where anthraquinones can come and bind. After binding, the pH drops to 5.5. At this pH, the self-assembly of casein micelles takes place by complexation with anthraquinones.

Moreover, these self-assembled CM hold the anthraquinones (AQ) tightly inside without any significant change in size as CM are tightly packed at pH 5.5 [54] through hydrogen, hydrophobic and electrostatic interactions [55]. It has been reported that bovine β-lactoglobulin (βLG) has a high affinity for binding anthraquinones through a change in secondary structure [37]. In addition, Moeller and colleagues [40] reported successful encapsulation of β-carotene, vitamin D_2_ or docosahexaenoic acid (DHA) into casein micelles at pH 5.5 without significant change in the size of CM.

Conversely, the average particle size of CM showed a dramatic increase (*p* = 1.00) from ~178 nm to ~279 nm with the encapsulation of FDP (CMFDP), while an increase from ~178 nm to 188 nm (*p =* 0.444) in case of SDP(CMSDP). This effect is due to the superficial binding of powders onto casein micelles. The binding of casein micelles and polyphenols depends upon their bonding type (hydrogen, hydrophobic, van der Waals), polyphenol type and structure, and protein–polyphenol ratio. In our study, the anthraquinones from SDP and FDP underwent dehydration stresses and, therefore, a low casein micelle: anthraquinones ratio that might not be high enough to form multiple noncovalent interactions, which are essential to obtain stable protein complexes [56]. Moreover, the distance among κ-casein may be broadened by the binding of anthraquinones. However, the significant increase in the size of CMFDP compared to CMSDP might be due to the formation of covalent bonds, which primarily occur upon the oxidation of anthraquinones to o-quinones or interactions with free cysteine residues on casein micelles [47].

In CMWLAG nanocapsules, agglomeration occurred even at low protein (4 g gel/g of casein micelles) and high anthraquinone concentrations. The binding sites of casein micelles were saturated with anthraquinones due to the high anthraquinone ratio, and larger aggregates formed by forming bridges between saturated casein micelles [57], which got precipitated. In the case of CMWLAG, the particle size of casein micelles could not be determined due to the sedimentation of the aggregated casein particles.

The average ξ-potential of encapsulated CMs with aloin (CMA), aloe-emodin (CMAE) and rhein (CMR) did not show any statistical difference (~−22 mV) as compared to the control (−23 mv). This indicated that anthraquinones were bonded inside the casein micelles and not attached to the surface of CMs [58]. Similar values have been reported previously, where CM was loaded with metformin, flutamide and quercetin [58,59,60], which have similar structures to anthraquinones. The ξ-potential with the addition of AQ powders was −17.56 ± 0.20 mV. This is due to the decrease in pH (5.5) after encapsulation. This pH shift corresponds to the decreased charge as the casein micelles are disrupted at pH 5.5 and the κ caseins no longer form an external negatively charged layer [61]. In addition, this decrease in negative charge suggested that AQ first binds to the core of CM mainly by hydrophobic interactions, as discussed previously. When the hydrophobic core is fully loaded, it starts electrostatically binding to the negatively charged CM surface, whose charge is dominated by the serine-phosphate groups in the hydrophilic N-terminal, increasing the zeta potential. These values were comparable to the earlier literature where CM cross-linked with genipin used to entrap alfuzosin hydrochloride drug [62]

ξ-potential values of CMFDP and CMSDP were −16.06 ± 0.50 and −16.03 ± 0.80, respectively, as compared to the control CM −23.73 ± 0.66 mV (Table 2). The decrease in negative charge suggested the successful coating of samples (FDP, SDP, WLAG) on the surface of the micelles instead of encapsulating them inside. This has also been depicted by comparing the ratio of anthraquinones in SDP and FDP, which is less than AQ. As the polyphenol/CM ratio decreased, the zeta potential value increased to −16 mV due to the positive charge on anthraquinones. Moreover, FDP and SDP bound electrostatically to negatively charged CM surface, dominated by serine phosphate groups in the hydrophilic terminal, increasing zeta potential as previously reported by [62].

**Figure 3:**
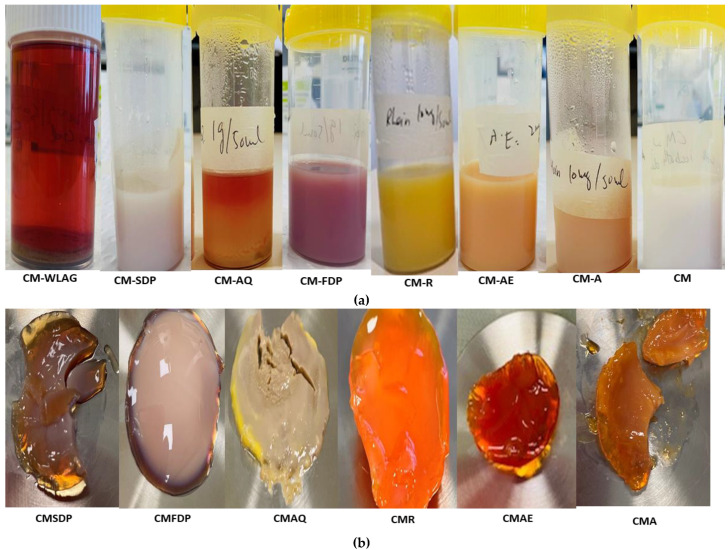
Visual observations of nanocapsules of CM controlled and CM-loaded with aloin (CMA), aloe-emodin (CMAE), rhein (CMR) and CM-loaded with freeze-dried powder (CMFDP), anthraquinones powder (CMAQ), spray dried powder (CMSDP) and whole leaf Aloe vera gel (CMWLAG) before centrifugation (**a**) and pallets of casein micelles nanocapsules after centrifugation (**b**).

Interestingly, the CMWLAG has the lowest negative charge (−11.96 ± 0.63) as pH is close to isoelectric point pI (pH 4.6), where hairy κ-casein is destabilized. So, CM precipitated out of the solution while making complex with WLAG at pH 4.6 (Table. 2) by releasing calcium phosphate into the solution. As the salt content of the solution increases, the electrical double layer is compressed and the zeta potential decreases. After a certain point, the electrical double layer collapses and becomes the same as the surrounding media, leaving the particles prone to agglomeration effects. The lower ξ-potential obtained might be due to the agglomeration of CM, as particles with zeta values between −10 mV and +10 mV experience rapid agglomeration unless they are sterically protected. As evidenced previously [63], the casein micelles with a slight net charge tended to aggregate at the isoelectric point. These results also are consistent with the previous literature [21,64,65]. Casein micelles can be seen as sediment on the bottom in nanocapsules of CMWLAG (Figure 3a).

### 2.4. Study of Casein Micelles-Anthraquinones Interactions by Fourier-Transform Infrared Spectroscopy (FTIR) for Nanocapsules

**Figure 4:**
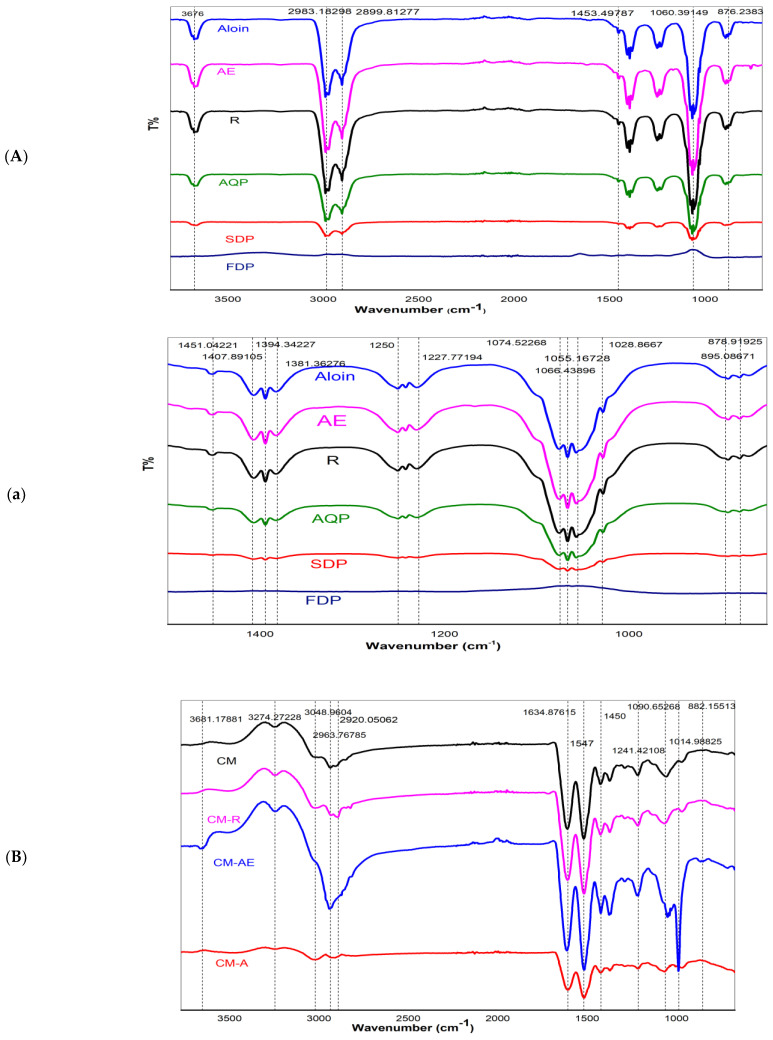

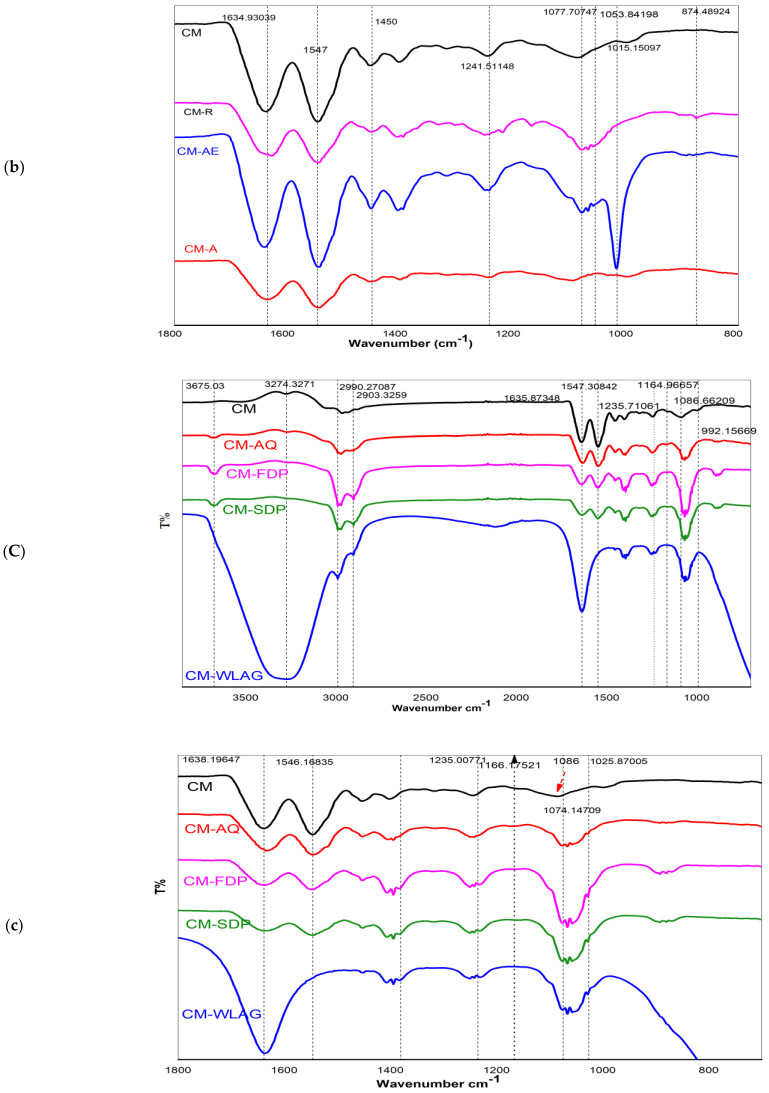
FTIR spectra of aloin (aloin), aloe–emodin (AE), rhein (R), Aloe vera extracted anthraquinones powder (AQ), spray–dried (SDP) and freeze-dried (FDP) powders of Aloe vera {(**A**) (3800–800 cm^–1^), (**a**) (1600–800 cm^–1^)}, controlled Casein micelles (CM) individual and its nanocapsules with pure compounds of aloin (CMA), aloe–emodin (CMAE), rhein (CMR) {(**B**) (3800–800 cm^–1^), (**b**) (1800–800 cm^–1^)}, Controlled casein micelles individual (CM) and its nanocapsules with various Aloe-vera powders (CMAQ, CMFDP, CMSDP, CMWLAG) {(**C**) (3800–800 cm^–1^), (**c**) (1800–800 cm^–1^)}.

Figure 4A,a shows infrared spectra of pure aloin, pure aloe-emodin (AE), pure rhein (R), anthraquinone (AQ) powder extracted from WLAG, freeze-dried powder (FDP) of aloe vera and spray-dried (SDP) powder of aloe vera. All samples generally exhibited a broad and intense absorption band at 3676 cm^−1,^ which can be attributed to the -OH stretching of anthraquinones (phenolic groups) in aloin, aloe-emodin and rhein [66]. These compounds contain hydroxyl groups directly attached to an aromatic hydrocarbon, which are pure compounds as apparent from their peaks. The anthraquinones extract from *aloe vera* (AQ) also showed strong -OH band-like standards suggesting the presence of anthraquinones in extracted powder. Conversely, in the case of FDP and SDP, the intensity of the -OH band was reduced considerably, probably due to the absence of anthraquinones [67] resulting from the deacetylation of bioactives [14]. Two bands at 2989 cm^−1^ and 2899 cm^−1^ are related to C-H symmetric stretching and asymmetric stretching of methylene (CH_2_), respectively, indicating long aliphatic chains (-CH) of anthraquinones [68] in samples (aloin, aloe-emodin, rhein, AQ). However, in FDP and SDP, a further decrease in -OH, C=C stretching bands corresponds to oxidation of aloin. The minor peaks at 1407 cm^−1^ and 1250 cm^−1^ are from the -COO of carboxylate compounds and C-O-C stretching of (COCH_3_), probably related to methyl acyl groups, indicating the presence of O-acetyl ester [69]. Peaks at 1055 cm^−1^ are associated with the C–O stretching of ester and phenols [70]. Two bonds between 800–900 cm^−1^ correspond to the C-H deformation of the pyranoside ring and mannose [71].

Figure 4B,b shows infrared spectra of rehydrated casein micelles (CM) and their nanocapsules with pure compounds of aloin, aloe-emodin and rhein (CMA, CMAE, CMR) at different wave numbers. Control CM had peaks at 3274 cm^−1^, 2903 cm^−1^ and 3001 cm^−1^ for -OH, CH_2_ and CH_3,_ respectively. Two vibration bands at 1634 cm^−1^ and 1547 cm^−1^ are related to amide I (C=O, C-N stretching) and II bands (N-H) [72], respectively. The anthraquinones-loaded CM exhibited altered bands in the frequencies and the intensities for -OH and amides. In anthraquinone-loaded casein micelles (CMA, CMAE, CMR; Figure 4B), amide Ι and amide Π appeared at1648 and 1547 cm^−1^ without any shift and the ester group (C-O-C) appeared at 1014 cm^−1^, while amine group C-N band appeared at 1090 cm^−1^ that sharpens in CMAE. This sharpness corresponds to hydrogen bond formation between the OH of anthraquinones and the carboxylic group of casein micelles [73]. However, in CMR, its transmittance is reduced due to fewer hydroxyl groups, leading to less hydrogen bond formation. As previously reported, a change in intensities at 3290, 2990, and 2963 cm^−1^ correspond to hydrophobic interactions [74], suggesting the methyl stretching of aliphatic chains of Anthraquinones. These hydrophobic interactions are due to the accommodation of anthraquinones’ aromatic rings on CM’s hydrophobic regions [75]. Therefore, it can be hypothesized that nanoencapsulation of casein micelles with pure anthraquinones (aloin, aloe-emodin, rhein) can be achieved by ultrasonication without changing casein micelles structure due to the stability of casein micelles, as previously reported [35,46]

Figure 4C,c shows IR spectra of control casein micelles (CM) and their nanocapsules with various aloe vera extracts (CMAQ, CMFDP, CMSDP, CMWLAG). The IR spectra of CM-loaded AQ powder (20 mg/mL) of Aloe-vera, its SDP and FDP (20 mg/mL each), as well as aloe-vera gel (WLAG) (4 g/mL), shows some similarities. Two peaks at 2990 and 2903 cm^−1^ are related to =C-H stretching vibrations and two characteristic peaks at 1635 and 1547 cm^−1^ were observed in CMAQ, CMSDP and CMFDP. No significant shifting of amide bands (I, II) occurred while encapsulating AQ, SDP and FDP, indicating that CM structure remained unchanged while forming complexes. In the case of interaction between CM and AQ, Figure 4c showed that the amine group (C-N) appeared at 1086 cm^−1^ in CM and decreased to 1074 cm^−1^ in CMAQ, corresponding to the hydrogen bond formation as discussed previously [22] but absent in CMFDP, CMSDP and CMWLAG. A change in intensities of CH_2_ bands at 2903, 2990 cm^−1^ correspond to hydrophobic interactions between CM and anthraquinones due to powerful polyphenols’ interaction with proline residues [76] in CMAQ, CMFDP, CMSDP and CMWLAG. The -OH stretching at 3290 cm^−1^ attributed to hydrogen bonds between casein micelles and AQ; however, these -OH bands were absent in CMFDP and CMSDP due to the absence of hydrogen bond formation between casein micelles as these have been present more on the surface, as discussed above. Interestingly, a broad-spectrum band at 3290 cm^−1^ was observed in WLAG, which is not related to casein micelles, but was due to water molecules of Aloe vera gel. Moreover, change in the amide II and disappearance of amide I band in CMWLAG is the indication of the deformation of the structure of casein micelles at isoelectric point *Ip* (pH 4.6), where caseins lose their emulsifying properties and precipitate.

## 3. Mechanism

The capability of casein micelles to encapsulate anthraquinones extracted from different *Aloe vera* samples which are sensitive to temperature, pH and oxidation [14] was investigated in the present study. Although casein micelles are cheap, sustainable and nutritious encapsulation vehicles for the protection of anthraquinones, it is necessary to understand the interactions and structural changes that occur during ultrasonication-assisted encapsulation.

**Figure 5:**
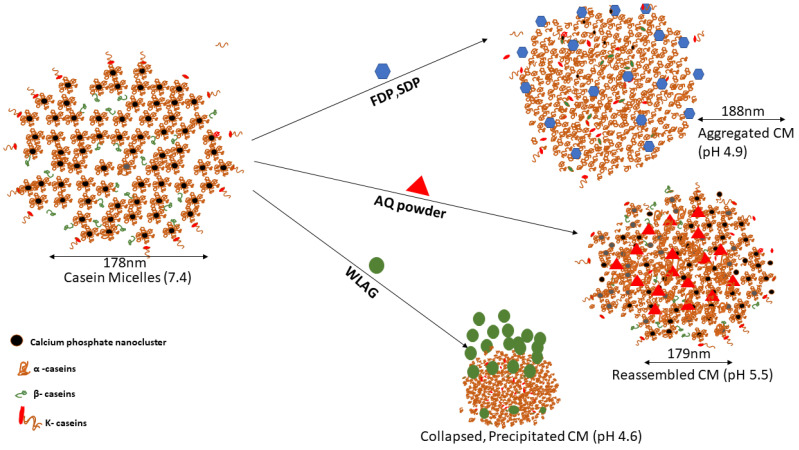
Schematic illustration of encapsulation mechanism of anthraquinones extracted from various samples of *Aloe vera* (AQ powder, FDP, SDP and WLAG).

The encapsulation efficiency and the physical, chemical and structural changes of encapsulated casein micelle mixtures depended mainly on the type of *Aloe vera* being encapsulated. Casein micelle is a dense protein network [45] where α-caseins and β-caseins are located in the inner part (Figure 5) while κ-caseins are attached to the surfaces [17]. It has been reported that there are four major interactions between casein micelles and bioactive components: hydrophobic interactions, hydrogen bonding, electrostatic interactions and van der Waals forces [35]. The higher encapsulation efficiency of AQ (98–100%) into casein micelles was due to the hydrophobic interactions, as casein micelles are capable of binding the lipophilic nutraceuticals stronger [77]. However, electrostatic interactions and hydrogen bonding were also present as per FTIR spectra where hydrogen bonding between the free hydroxyl groups and carbonyl groups was evidenced. Similarly, it has been reported that hydrophobic forces played a major role in casein micelle-emodin, α-tocopherol-caseins and EGCG-casein micelles [35,77]. Moreover, ultrasonication enhanced the binding of anthraquinones inside the casein micelles by hydrophobic interactions. Lower encapsulation efficiencies of FDP and SDP [14] were related to their accommodation on the hydrophilic surface of CM (superficial attachment) through hydrogen bonding during encapsulation.

pH also plays an essential role in determining encapsulation efficiency. The addition of AQ powder reduced the pH of casein micelles solution to 5.5. As previously discussed, the casein micelles joined together through hydrogen bindings and electrostatic and hydrophobic interactions. A decrease in pH causes a reduction in the negative charge of casein micelles and a slight dissolution of calcium phosphate which forms individual calcium and phosphate ions. The pH-induced reorganization of CM structure led to shrinkage of casein micelles, and Ca and phosphate ions were further displaced from CM by hydronium ions. The resulting re-assembled CM holds the entrapped AQ tightly within a hydrophobic core (Figure 5) without a significant change in size and resulting in higher encapsulation efficiencies and yield for aloin, aloe-emodin and rhein (Figure 2c). The rearrangement of CM was confirmed by no change in the amide I and amide II bands in FTIR spectra. The low encapsulation yields with SDP and FDP were due to hydrophobic precipitation of CM and the drastic increase in particle size was due to the aggregation of CM (Figure 5) at acidic pH (4.8–4.9; Table 2). An increase in particle size corresponds to decreased physical stability where unstable nanoparticles are more likely to show low encapsulation efficiencies. On the contrary, encapsulating whole leaf aloe vera gel (having natural pH of 4.52), which contains almost 90% of water, caused a decrease in overall pH to 4.6, where complete solubilization of calcium phosphate nanoclusters takes place. The isoelectric point of CM is 4.6 and CM is capable of self-assembling and aggregating due to the loss of electrostatic repulsion among them [78]. This destabilization of the CM structure caused a decrease in encapsulation efficiency.

The present study confirmed that anthraquinones can be protected from degradation through heat, light and oxidation preferably encapsulated in the form of AQ powder produced from Aloe vera leaf into casein micelles. It has been previously recommended that the daily dose of anthraquinones should be 10 ppm in functional foods and health drinks as per International Aloe Science Council (IASC) [79]. So, 10 mg of anthraquinones in pure form and 1 g in the case of *Aloe vera* extracted anthraquinones powder (AQ) would provide the daily requirement if it got encapsulated into 2% casein micelles solution.

## 4. Conclusions

In the present study, casein micelles were successfully used to encapsulate anthraquinones powder (AQ) and pure aloin, aloe-emodin and rhein through ultrasonication. Anthraquinones powder (AQ) was extracted from *Aloe vera* gel after evaporation of extraction solvent followed by freeze drying to obtain maximum stability. This AQ powder had the maximum encapsulation efficiency and yield compared to SDP, FDP and WLAG, based on anthraquinones: casein micelles ratio, the structure of anthraquinone, and pH of the solution. FTIR spectra indicated that AQ powder binds to CM without affecting its structure, while WLAG caused the precipitation of CM. Overall, these results show that it is possible to supply a recommended dose of anthraquinone (10 ppm) by adding 1 g of extracted anthraquinone powder to 1 g of casein micelles, and their size and zeta potential suggest enough colloidal stability for incorporation into new functional foods and nutraceuticals. More studies are required to determine the stability of casein micelles-loaded nanocapsules, and their influence on human physiological functions such as digestion and bioavailability.

## 5. Materials and Methods

### 5.1. Materials

Casein micelles powder was procured from reliable resources. Aloe vera (*Aloe barbadensis*) leaves were collected from Aloe vera Australia (Goodman International, Brisbane, Australia). Whole leaf *Aloe vera* gel extracts and their spray-dried and freeze-dried powders were prepared by the method described previously [14]. Standards of Aloin (Mikromol, item code: MM1318.01, CAS# 1415-73-2), Aloe-emodin (TRC, item code: A575400-10MG, CAS #481-72-1), and Rhein CRS (EDQM, item code: EPY0002159, CAS# 478-43-3) were purchased from Novachem pty Ltd. (25 Carissane Road, Heidelberg West, Victoria, Australia). HPLC grade methanol was purchased from Sigma Aldrich Pty Ltd. (Castle Hill, New South Wales, Australia). MilliQ water was used at all times.

### 5.2. Sample Preparation

#### 5.2.1. Anthraquinones Extraction

*Aloe vera* gel extracted anthraquinone powder (AQ) was prepared by centrifuging WLAG at 6000 rpm for 20 min using the Beckman centrifuge (Allegra 64R-Beckman Coulter, Chaplin Drive, Lane Cove West, New South Wales, Australia). The (supernatant) clear gel was collected, and methanol was added in the ratio of 50:50, followed by sonication for 10 min using an ultrasonic bath (FXP12, Unisonics, Wattle Rd, Brookvale, New South Wales, Australia) at ~4 ± 1℃ to extract anthraquinones. After the extraction process, solvent evaporation was performed by the Rotational Vacuum Concentrator (Model # RVC2-33CD Plus, John Morris Scientific Pty Ltd., Victoria Avenue, Chatswood, New South Wales, Australia) for eight hours at 15,000 min^−1^ rotation and a temperature of 25 °C to obtain the methanol-free extract. The remaining liquid (water in gel) in the extract was removed using a freeze dryer (Labconco FreeZone Triad Benchtop Freeze Dryer, Prospect Avenue, Kansas City, MO, USA) to obtain brown-colored anthraquinone powder extracts (AQ). Extraction and quantification of anthraquinones were performed in HPLC by the method given in the previous article [14].

#### 5.2.2. Preparation of Casein Micelle Solutions

Casein micelle solutions with a protein concentration of 2% *W*/*W* were prepared in MilliQ water. The casein micelle solution was stirred continuously (300 rpm) by a magnetic stirrer (9 MR Hei-Tec Stirrer + Pt1000 V4A) at a temperature of 50 °C for 1 h, followed by stirring at room temperature for an additional 3 h. Casein micelles were allowed to rehydrate in the refrigerator at 4 °C for one day. The solutions were stirred and equilibrated at 25 °C before encapsulation.

#### 5.2.3. Encapsulation of Anthraquinones into Casein Micelles

*Aloe vera* extracted anthraquinones from WLAG, AQ, SDP and FDP and pure aloin, aloe-emodin and rhein compounds were encapsulated. Concentrations of 0.02 mg/mL of pure compounds, 20 mg/mL of AQ, SDP and FDP and 4 g/mL of WLAG were used. The encapsulation process started by sonicating casein micelle-anthraquinone mixtures using the 20 kHz ultrasound equipment (500 W) operating at 50% amplitude by placing the 12.7 mm ultrasonic probe at the center of the sample at 2 cm depth. The temperature was maintained at 25 °C by placing an ice tub under the sample beaker. The sonication was set at 15 min with 30 s pulse on time and 30 s pulse off-time. The actual power delivered to the solution was 39.74 W, as determined by calorimetry. Ultrasonicated casein micelle solution without added anthraquinone compounds was considered the control for comparison purposes.

### 5.3. Characterization of Nanocapsules

#### 5.3.1. Determination of Encapsulation Efficiency

The freshly prepared anthraquinone-loaded capsules were centrifuged at 19,000 rpm for 90 min at 20 °C (centrifuge 3-30kHS-Sigma) to precipitate the micelles. The supernatant was recovered and the anthraquinone concentrations were quantified as per the method given previously [14] using an Agilent series1220 infinity gradient HPLC (Agilent Technologies, CA, USA) equipped with a 600 solvent pump and a C_18_ reversed-phase packing column (Phenomenex XB-C18, 4.60 mm × 250 mm, Aeris Wide pore 3.6 μm). Free Anthraquinones were detected at 254 nm and quantified against an established calibration curve. Encapsulated anthraquinones were determined as the difference between the initial concentration added and the quantified concentration in the supernatant obtained after centrifugation, whereas encapsulation yield correlates with the milligrams of anthraquinones encapsulated per gram of protein (casein micelles). The formula (1) and (2) were used to determine encapsulation efficiency (EE) and encapsulation yield (EY), respectively.

(1) EE (%)=milligrams of anthraquinones encapsulated/milligrams of anthraquinones added ×100

(2) EY (mg/g)=milligrams of anthraquinones encapsulated/grams of casein micelles in solution

#### 5.3.2. Particle Size Distribution and Zeta-Potential Measurements

The particle size and zeta potential of anthraquinone-loaded casein micelles were obtained using the ZetaSizer Nano ZS (Malvern Instrument Ltd., Worcestershire, UK). Three measurements were taken at ambient temperature (25 ± 1 °C) from 12 runs per measurement. The samples were diluted at 1:10 fold in water and analyzed, employing a refractive index of 1.57 for the sample and 1.33 for the dispersant

#### 5.3.3. FTIR Spectroscopic Analysis

FTIR spectra were obtained in transmittance mode in the 4000–400 cm^−1^ wavelength range using GladiATR-Fourier transform infrared spectroscopy (Perkin Elmer Pike Technologies, Treble Cove Rd. Billerica, MA, USA) to evaluate the interactions of casein micelles and anthraquinones. The measurements were carried out for control casein micelles (CM), individual samples of anthraquinones (aloin, aloe-emodin, rhein, AQ, FDP, SDP, WLAG), as well as casein micelles loaded with aloin (CMA), aloe-emodin (CMAE), rhein (CMR), anthraquinone powder (CMAQ), spray, dried powder of *Aloe vera* (CMSDP), freeze-dried powder of *Aloe vera* (CMFDP) and whole leaf *Aloe vera* gel (CMWLAG). Sixteen scans were performed, and the resolution used was 4 cm^−1^. Full FTIR spectra were recorded in triplicate for each sample, and the average spectrum was obtained.

#### 5.3.4. Statistical Analysis

All measurements were performed in triplicates, and results were expressed in means ± standard deviation. Statistical differences between treatments were evaluated by one-way analysis of variance (ANOVA) available in software SPSS statistical software (23.0 version, Michigan State University, East Lansing, MI, USA). Duncan’s multiple range test was used for mean separation and to define significance differences (*p* < 0.05). Encapsulation efficiency and yield were created on Graph pad Prism 9.1.0(221) (GraphPad Software LLC, Inc., San Diego, CA, USA), and *p-*value < 0.05 was considered statistically significant. Also, FTIR figures were created using OriginPro 8.0 software (OriginLab Corporation, Northampton, MA, USA).

## Figures and Tables

**Table 1:** Concentrations of Anthraquinones in WLAG, SDP, FDP and AQ powder.

Sample (mg/g of Dry Powder)	AQ	WLAG	FDP	SDP
Aloin	14.78 ± 0.46 ^a^	6.16 ± 0.01 ^a^	0.223 ± 0.0 ^b^	0.220 ± 0.2 ^b^
Aloe-emodin	5.73 ± 0.29 ^a^	0.051 ± 0.00 ^c^	0.00	0.018 ± 0.0 ^c^
Rhein	2.078 ± 0.03 ^a^	0.00	0.00	0.00

Values are mean and standard deviations from triplicate data. The data was analysed by one-way ANOVA and Tukey’s post hoc HSD tests. The different letters in superscript (a, b, c) within rows indicate statistically significant differences (*p* < 0.05).

**Table 2:** Mean particle size and zeta potential of controlled casein micelles (CM); with sonication) and casein micelles-loaded with anthraquinones (CM-aloin, CMAE (aloe-emodin), CMR (rhein), CMAQ (anthraquinones), CMFDP (freeze-dried powder), CMSDP (spray dried powder), CMWLAG (whole leaf aloe vera gel).

Samples Name	Particle Size (nm)	ξ Potential (mV)	Observed pH
CM Control	177.9 ± 0.70 ^cd^	−23.73 ± 0.66 ^a^	7.48 ± 0.03
CM-Aloin	173.86 ± 1.26 ^f^	−22.6 ± 0.15 ^ab^	7.45 ± 0.04
CM-AE	175.36 ± 0.37 ^de^	−22.2 ± 0.17 ^b^	7.44 ± 0.05
CM-R	171.5 ± 2.97 ^f^	−22.73 ± 0.55 ^ab^	7.40 ± 0.07
CM-AQ	179.16 ± 1.12 ^c^	−17.56 ± 0.20 ^c^	5.52 ± 0.03
CM-FDP	278.86 ± 2.90 ^a^	−16.06 ± 0.50 ^d^	4.90 ± 0.07
CM-SDP	188.36 ± 1.9 ^b^	−16.03 ± 0.80 ^d^	4.81 ± 0.07
CM-WLAG	ND	−11.96 ± 0.63 ^e^	4.61 ± 0.03

Values are mean and standard deviations from triplicate data. The data was analysed by one-way ANOVA and Tukey’s post hoc HSD tests. The different letters in superscript (a, b, c, ab, d, cd, e, de, f) within rows indicate statistically significant differences (*p* < 0.05).

## Data Availability

The data presented in this study are available on request from the corresponding author.
